# Optimizing Osseointegration in Dental Implantology: A Cross-Disciplinary Review of Current and Emerging Strategies

**DOI:** 10.7759/cureus.47943

**Published:** 2023-10-30

**Authors:** Turki Abu Alfaraj, Sarah Al-Madani, Nadeen S Alqahtani, Abdulrhman A Almohammadi, Albatool M Alqahtani, Haifaa S AlQabbani, Mohammed K Bajunaid, Badr A Alharthy, Norah Aljalfan

**Affiliations:** 1 Periodontics, National Guard Hospital, Medina, SAU; 2 Periodontics, Saudi Ministry of Health, Riyadh, SAU; 3 Dentistry, Saudi Ministry of Health, Jeddah, SAU; 4 Dentistry, Alhomaidi Medical Complex, Medina, SAU; 5 Dentistry, Saudi Ministry of Health, Khamis Mushayt, SAU; 6 Pediatric Dentistry, Al Yamamah Hospital, Riyadh, SAU; 7 Dentistry, King Abdulaziz University, Jeddah, SAU; 8 Dentistry, King Abdulaziz University Hospital, Jeddah, SAU; 9 Dentistry, Saudi Ministry of Health, Dammam, SAU

**Keywords:** implant design, tissue engineering, implant stability, osteointegration, implant placement

## Abstract

The paper explores the correlation between osteointegration and dental implant stability, investigating the relationship and its implications for successful outcomes in implant dentistry. Osteointegration, defined as the direct structural and functional connection between living bone and the implant surface, plays a crucial role in determining the stability and long-term success of dental implants. This review synthesizes current knowledge from scientific literature and clinical studies to elucidate the factors influencing osteointegration and their impact on implant stability. Surface characteristics of implants, such as topography and chemistry, as well as the surgical techniques employed during implant placement, are examined in detail, emphasizing their significant influence on osseointegration and subsequent implant stability. Additionally, host-related factors such as bone quality, systemic conditions, and patient-specific considerations are explored to further comprehend the complexity of the osteointegration process. The abstract underscores the importance of achieving an optimal bone-implant interface to ensure successful implant integration and stability. Furthermore, emerging technologies and materials, such as computer-guided implant placement and biomimetic surfaces, are discussed for their potential to enhance osteointegration and improve long-term implants.

## Introduction and background

Dental implants have revolutionized restorative dentistry by providing an effective solution for replacing missing teeth. Compared to traditional prosthetic options such as bridges or dentures, dental implants offer a more permanent and natural alternative [1]. These implants are made of biocompatible materials, like titanium, and are surgically placed into the jawbone to serve as artificial tooth roots. Over time, the implant surface fuses with the surrounding bone in a process called osseointegration, which establishes a stable foundation for prosthetic restoration [2].

Dental implants' long-term success depends on their stability within the oral environment. Implant stability has a direct impact on the functionality, aesthetics, and overall satisfaction of patients with implant-supported restorations [3]. Stable dental implants provide secure anchorage, ensuring effective chewing and speaking abilities [4]. Additionally, they help maintain the integrity of the surrounding bone and soft tissues [5].

Osseointegration is the process of creating a strong bond between living bone and the surface of an implant [2]. This is accomplished by forming new bone tissue that surrounds and attaches to the implant, providing the necessary mechanical stability. The success of osseointegration depends on several factors, such as implant design, surface characteristics, surgical techniques, and host-related factors [3]. The stability of dental implants and their long-term success are directly related to the degree of osseointegration achieved. To ensure predictable and durable outcomes in implant dentistry, it is essential to achieve optimal osseointegration [3].

The primary objective of this study is to undertake a comprehensive examination of the various techniques and approaches in dental implantology that contribute to enhanced osseointegration. A significant focus is placed on deciphering the influence of nanostructured titanium surfaces on bone formation and the enzyme alkaline phosphatase (ALP). Concurrently, the research delves into the merits and challenges presented by both physical and chemical modification techniques applied to titanium surfaces. There's also an exploration into the potential benefits of pharmaceutical and nutritional interventions, with substances such as bisphosphonates, melatonin, and vitamin D, all of which play critical roles in the osseointegration process. In addition, the transformative potential of tissue engineering strategies, especially the deployment of mesenchymal stem cells (MSCs), platelet-rich plasma (PRP), and vascular endothelial growth factor (VEGF), will be assessed in the context of promoting bone regeneration around dental implants. The study further examines the advancements brought forth by 3D printing technology in crafting dental implants and scaffolds, aiming to draw a holistic picture of the current state and future prospects of dental implantology.

## Review

Search methodology

Using PubMed, Google Scholar, Embase, and MEDLINE databases for the keywords “Implant osteointegration, osteointegration, Titanium osteointegration, Zirconia osteointegration,” we retrieved all clinical trials and systemic reviews and meta-analyses. Only studies that were written in English were included. Inappropriate articles by title were omitted. Further exclusion by reading the abstract led to the most appropriate articles for our topic.

Osteointegration process

Definition and Stages of Osteointegration

Osteointegration involves a series of cellular and molecular events that lead to a direct structural and functional connection between the implant surface and the bone [6]. The osteointegration process can be divided into four stages. The first stage is called the Initial healing stage; this stage occurs immediately after implant placement. The implant comes into contact with the surrounding bone and a blood clot forms around the implant's surface. Within a few hours, platelets and inflammatory cells migrate to the implant surface, initiating the inflammatory response [7]. The second stage, called Inflammatory stage inflammation, triggers the recruitment of various cells to the implant site, including neutrophils and macrophages. These cells remove debris, bacteria, and damaged tissue. The inflammation also stimulates the release of growth factors and cytokines, which are crucial in subsequent healing processes. The third stage is the Proliferative stage. During this stage, osteoblasts, which are bone-forming cells, begin to migrate and proliferate in the surrounding bone. They deposit a new bone matrix around the implant surface. The osteoblasts gradually form a mineralized matrix, bridging the implant and the bone [7]. The last stage is the Remodeling stage. In this final stage, the newly formed bone undergoes remodeling, which involves the removal of old and damaged bone and replacing new mature bone. The remodeling process helps to strengthen the bone-implant interface further and optimize the mechanical properties of the integrated implant [7].

Cellular and Molecular Events During Osteointegration

The osteointegration process is influenced by various cellular and molecular events that collectively contribute to its success. One important event is angiogenesis, where new blood vessels form to deliver oxygen, nutrients, and cells to the implant site. This early-stage occurrence establishes the necessary vascular supply for subsequent healing processes. Another critical event is osteoblast differentiation and bone formation [8]. Osteoblasts, derived from mesenchymal stem cells, play a key role in creating new bones. Growth factors and signaling molecules, such as bone morphogenetic proteins (BMPs) and transforming growth factor-beta (TGF-β), stimulate the differentiation of mesenchymal stem cells into osteoblasts. These osteoblasts produce a collagen-rich matrix, which eventually mineralizes and becomes new bone [8].

Additionally, osteoclast activity is vital during the remodeling stage of osteointegration. Osteoclasts specialize in bone resorption, breaking down old or damaged bone. This process allows room for osteoblasts to deposit new bone, ensuring bone quality maintenance and facilitating adaptation at the bone-implant interface [8].

Different Approaches to Enhance Dental Implant Osseointegration

Implants: Surface Characteristics, Modifications, and Their Impact on Bone Integration

Engineering studies have demonstrated that the biocompatibility, shape, and design of an implant, along with its surface characteristics, have a significant impact on the osseointegration of the implant [9]. The surface characteristics of the implant play an essential role in either aiding or hindering the integration of bone by affecting protein and cell interactions [10]. A study conducted by Zhu et al. classifies various surface modification approaches into four categories: mechanical modification, physical modification, chemical modification, and biological modification [10].

Mechanical modification methods such as grinding, polishing, sandblasting, and vacuum annealing are employed to alter the surface conditions of biomaterials, including dental implants, from smooth to rough [11]. These techniques significantly influence various surface properties, including biological adhesion, hydrophilicity, affinity for bone tissue, electrical potential energy, and surface tension. The induced surface roughness can be precisely categorized into macro, micro, and nano scales, each having distinct implications for the material's interaction with its environment and overall performance in dental applications.

Macroroughness in dental implants refers to surface irregularities on a larger scale, typically ranging from millimeters to micrometers. It is directly related to the implant's overall design and structure, including macroscopic features like threads and porous structures, which contribute to initial implant stability by enhancing primary fixation [12]. Microroughness, characterized by smaller-scale surface irregularities typically at the micrometer level, is achieved through techniques like sandblasting and acid-etching. This level of texture plays a pivotal role in the success of dental implants. By facilitating better adhesion and growth of bone cells, microroughness is crucial for osseointegration, directly contributing to the long-term stability and overall success of dental implants.

Nanoroughness, distinct from microroughness, is a defining surface characteristic of dental implants. It is characterized by minuscule irregularities measuring less than 100 nanometers. This fine-scale roughness is not merely a structural peculiarity, it profoundly affects the osseointegration process. Studies have indicated that nano-rough surfaces bolster osseointegration by creating a favorable microenvironment for the proliferation of bone-forming osteoblasts, thereby enhancing bone-to-implant contact. These surfaces also encourage increased protein adsorption, integrating crucial molecules like fibronectin and vitronectin [13]. A significant advantage of nanoroughness is its ability to resist bacterial adhesion and biofilm formation, substantially reducing the risk of peri-implantitis. Both micro and nanoroughness on titanium surfaces play pivotal roles in various aspects of bone-titanium integration, affecting parameters such as alkaline phosphatase (ALP) activity, the strength of integration, osteoconductivity, and the number of osteoblasts attaching to these surfaces [14].

ALP is an enzyme that supports bone formation. Recent studies have shown that nanostructured titanium surfaces can enhance ALP activity in osteoblasts, promoting their differentiation and mineralization processes. This suggests that nanoroughness positively impacts ALP activity, which facilitates bone healing around titanium implants [14].

The strength of bone-titanium integration is crucial for implant stability. Nano-scale roughness enhances integration by increasing the contact area between the bone and titanium, ultimately improving the mechanical stability of implants. This interlocking effect greatly contributes to the long-term success of implants, ensuring that they remain firmly anchored in the bone tissue [14].

Osteoconductivity refers to the ability of a material to support bone ingrowth, while osseointegration is the direct structural connection between bone and implant surfaces. Nanostructured titanium surfaces, with their increased surface area and favorable topography, promote osteoconductivity and enhance osseointegration. These surfaces provide an ideal environment for bone cells to attach, proliferate, and form a robust bond with the implant, ultimately contributing to its long-term success [15].

The number of osteoblasts attached is also important. Micro- and nano-scale roughness increases the available osteoblast attachment sites on titanium surfaces, leading to more osteoblasts attaching to the implant. This is essential for initiating bone-titanium integration and improving implant stability and successful integration [15].

Physical alteration techniques involve modifying surfaces with minimal or no chemical reactions. These techniques include plasma spraying technology (PST), plasma immersion ion implantation (PIII), laser cladding, and hydroxyapatite (HA) coatings [10]. PST uses a plasma arc to ionize inert gas, creating a high-temperature plasma flame. This flame melts or semi-melts materials like ceramics, metals, and alloys, which are then sprayed onto a pre-treated implant surface to create a durable coating. PST has the advantages of a fast deposition rate, large deposition thickness, and low cost [10].

HA coatings, which are used in clinical settings to promote osseointegration, are created by spraying HA particles onto the implant surface at high temperatures and quickly cooling them. However, HA coatings suffer from certain drawbacks, with a key limitation being their strong attachment to bone tissue but relatively weak bonding to the underlying metal alloy substrate [16].

Chemical modification techniques typically involve intense chemical reactions, especially when applied to a titanium (Ti) substrate. This occurs at the junction of the metal surface and the surrounding medium, whether it is a liquid solution or gas. This process often involves luminescence, heating, redox reactions, and other effects. Methods such as sandblasting and acid etching, thermal oxidation, hydrothermal treatment, anodic oxidation, and micro-arc oxidation have been extensively used to create surfaces with unique topographical structures and intricate compositions [10].

Anodic oxidation (AO) is a widely used method for modifying metal surfaces by creating oxide films through electrochemical oxidation. In this process, the metal is secured to the anode of an electrolytic cell. When a specific voltage is applied, ions in the electrolyte migrate to the cathode and anode due to the electric field, leading to redox reactions. This results in a continuous cycle of oxide film formation and removal, ultimately producing a distinct surface structure characterized by micro/nanoscale pore or tube arrays. AO can be applied to modulate surface charge, which is important since electrical stimulation can promote osteogenesis through biological interactions, such as with naturally charged biomolecules [10].

Pharmaceutical Interventions in Dental Implant Osseointegration

Several studies have shown that the use of local pharmaceuticals can improve the integration of implants with bone [17]. Different types of pharmaceuticals and dietary supplements, such as bisphosphonates, antimicrobial medications, and supplements to reduce inflammation, have varying degrees of effectiveness when administered locally. Some are very effective, while others have little to no effect on the process of osseointegration [18].

Bisphosphonates are a type of pharmaceutical that can reduce the activity of osteoclasts, which are responsible for bone resorption. When applied locally, such as to dental implants, they can improve the process of osseointegration [19]. Alternatively, an analysis by Alenezi and Chrcanovic found that with regard to the use of local antimicrobial agents during implant placement surgery, there was no significant effect in osseointegration [20].

Melatonin is a bioactive compound that has been shown to have a significant impact on bone remodeling and neoformation. It enhances osteogenic gene expression, leading dental pulp-derived MSCs (DPSCs) to differentiate into osteoblasts, essential for bone formation. Melatonin can also reduce bone resorption by inhibiting osteoclasts, which helps maintain bone density. Additionally, melatonin promotes BMSC osteogenic differentiation, aiding bone formation and mitigating bone loss [21]. These findings suggest potential applications of melatonin in dental treatments. In a study by Guardia et al. [22], the application of melatonin notably increased inter-thread bone volume and new bone formation compared to control implants lacking melatonin administration.

Vitamin D, when applied to the surface of dental implants, acts as a potent biological agent influencing osseointegration. Receptors in osteoblasts responsive to vitamin D directly modulate cellular activities by controlling gene expression. In conjunction with key proteins essential for bone formation, like osteocalcin, these receptors are impacted by vitamin D, thereby playing a significant role in maintaining bone health [23]. A controlled animal study conducted by Salomo-Coll et al. [24] revealed that the topical application of vitamin D during immediate implant procedures might have a minimal effect on osseointegration. However, dental implants with topical vitamin D treatment showed a marked decrease in crestal bone loss and an approximate 10% increase in bone-to-implant contact [24].

Statin drugs, commonly prescribed for cholesterol management, have garnered interest in the field of dental implantology for their potential to enhance osseointegration. Research indicates that statins promote bone formation by upregulating the expression of bone morphogenetic protein-2 (BMP-2), a key regulator in bone metabolism and regeneration. A study by Jun et al. [25] revealed that the local application of simvastatin in a 25 mm dosage used as a gel around dental implants significantly increased bone-implant contact by 20% compared to control groups in a rabbit model.

Tissue Engineering Approaches: Harnessing MSCs, PRP, and VEGF for Enhanced Osseointegration

Tissue engineering is a significant development in regenerative medicine. It combines cell biology, materials science, and engineering principles to restore, maintain, or enhance tissue functionality [26]. In dental implants, tissue engineering aims to improve osseointegration by promoting bone regeneration in the peri-implant region.

Regenerative dentistry has seen significant advancements with the strategic implementation of mesenchymal stem cells (MSCs), platelet-rich plasma (PRP), and the novel manipulation of vascular endothelial growth factor (VEGF). These methodologies help strengthen osseointegration, a critical requirement for the success of dental implant procedures.

MSCs have self-renewal and multipotent characteristics that benefit tissue repair and regeneration [27-28]. Bone marrow-derived MSCs (BMSCs) are readily accessible and tend towards osteogenic differentiation. On the other hand, umbilical cord MSCs (UCMSCs) have advantages such as higher proliferation rates and elongated telomeres, which suggest potentially advanced regenerative abilities.

Platelet-rich plasma (PRP) is enriched with critical growth factors, specifically platelet-derived growth factor (PDGF), TGF-β, and VEGF, which play pivotal roles in bone regenerative processes. These bioactive molecules participate in various regenerative activities, including chemotaxis, cellular proliferation, and angiogenesis [29]. In line with this, a randomized clinical trial by Kapoor et al. [30] demonstrated that platelet-rich fibrin (PRF) substantially enhances the osseointegration of dental implants in the initial phases of recovery.

Vasculogenesis is essential for the effective bonding of dental implants to bone tissue and for bone regeneration. Vascular VEGF, pivotal in vasculogenesis, plays a dual role by promoting the development of new blood vessels and supporting bone repair. Despite its recognized significance in these processes, the specific impact of VEGF on dental implants is yet to be fully explored. A study involving VEGF-coated implants showed encouraging results, indicating a marked increase in the proliferation of both osteoblasts and endothelial cells, leading to enhanced implant-bone integration and regeneration [31].

However, using MSCs, PRP, and VEGF for therapeutic purposes is full of challenges. The administration of VEGF, in particular, requires careful modulation to avoid excessive blood vessel formation, which could lead to detrimental consequences such as abnormal tissue expansion or subpar bone quality. Therefore, while the combined use of MSCs, PRP, and VEGF-coated implants presents new opportunities for dental regenerative medicine, it also requires thorough research to standardize protocols and evaluate long-term efficacy and safety.

The Revolution of 3D Printing in Dental Implantology and Tissue Regeneration

The emergence of 3D printing technology, also known as additive manufacturing, has significantly changed dental implantology and tissue engineering. This advanced process allows for the customization of implants and scaffoldings with high precision and reproducibility, which was previously unattainable through traditional manufacturing methods. By printing complex structures layer by layer, 3D printing enables the creation of intricate designs that closely match the patient's unique anatomical features. This has the potential to improve the fit and integration of dental implants [32]. Additionally, 3D-printed bioactive scaffolds can be excellent platforms for cell attachment and proliferation, especially when combined with MSCs, PRP, or growth factors such as VEGF. These scaffolds support cellular activities and gradually degrade to make room for newly formed tissue, thus playing a vital role in the guided regeneration of bone tissue [33]. However, to bring 3D-printed implants and scaffolds to the clinic, thorough research must evaluate their biocompatibility, mechanical strength, and long-term interaction with the biological environment.

## Conclusions

In the field of dental implantology, it's evident that the journey toward achieving optimal osseointegration is multifaceted. The careful selection of implant surface characteristics, coupled with informed choices regarding pharmaceutical interventions, underscores the importance of a comprehensive approach. The introduction of advanced techniques, specifically tissue engineering and 3D printing, represents a significant stride in aligning patient-specific needs with innovative solutions. The convergence of these techniques, from the microscopic modifications of implant surfaces to the potential of bioactive 3D-printed scaffolds, illuminates a promising horizon for the future of dental implants. However, as we navigate these advancements, it remains crucial to prioritize rigorous research and patient safety at each step.
